# SGS3 Cooperates with RDR6 in Triggering Geminivirus-Induced Gene Silencing and in Suppressing Geminivirus Infection in *Nicotiana Benthamiana*

**DOI:** 10.3390/v9090247

**Published:** 2017-09-04

**Authors:** Fangfang Li, Yaqin Wang, Xueping Zhou

**Affiliations:** 1 State Key Laboratory for Biology of Plant Diseases and Insect Pests, Institute of Plant Protection, Chinese Academy of Agricultural Sciences, Beijing 100193, China; elva1988@163.com; 2 State Key Laboratory of Rice Biology, Institute of Biotechnology, Zhejiang University, Hangzhou 310058, China; wangyq0219@126.com

**Keywords:** RNA silencing, geminivirus, RDR6, SGS3, resistance

## Abstract

RNA silencing has an important role in defending against virus infection in plants. Plants with the deficiency of RNA silencing components often show enhanced susceptibility to viral infections. RNA-dependent RNA polymerase (RDRs) mediated-antiviral defense has a pivotal role in resistance to many plant viruses. In RDR6-mediated defense against viral infection, a plant-specific RNA binding protein, Suppressor of Gene Silencing 3 (SGS3), was also found to fight against some viruses in *Arabidopsis*. In this study, we showed that *SGS3* from *Nicotiana benthamiana* (*NbSGS3*) is required for sense-RNA induced post-transcriptional gene silencing (S-PTGS) and initiating sense-RNA-triggered systemic silencing. Further, the deficiency of *NbSGS3* inhibited geminivirus-induced endogenous gene silencing (GIEGS) and promoted geminivirus infection. During TRV-mediated *NbSGS3* or *N. benthamiana RDR6 (NbRDR6)* silencing process, we found that their expression can be effectively fine-tuned. Plants with the knock-down of both *NbSGS3* and *NbRDR6* almost totally blocked GIEGS, and were more susceptible to geminivirus infection. These data suggest that NbSGS3 cooperates with NbRDR6 against GIEGS and geminivirus infection in *N. benthamiana*, which provides valuable information for breeding geminivirus-resistant plants.

## 1. Introduction

RNA silencing is a surveillance system in eukaryotic organisms induced by double-stranded RNA (dsRNA) that is subsequently processed by a dsRNA-specific RNase III enzyme (Dicer) into 21–25-nucleotide (nt) long, small interfering RNA (siRNAs). Then, the siRNAs are loaded into the RNA-induced silencing complex (RISC), including ARGONAUTE (AGO) proteins, to guide the sequence-specific degradation of complementary RNAs [1,2,3]. Several host RNA-dependent RNA polymerases (RDRs) in plants have been shown to be able to boost the generation of secondary siRNAs, which amplify defense signals [4]. In RDRs-mediated RNA silencing process, Suppressor of Gene Silencing 3 (SGS3), which accompanies with RDR6, is required to convert single-stranded to double-stranded RNA (dsRNA) to produce both exogenous and endogenous siRNAs [5,6,7]. SGS3 binds and stabilizes RNA templates during the initiation of RDR6-mediated dsRNA synthesis in *Arabidopsis* [8,9], and SGS3 co-localizes with RDR6 in SGS3/RDR6-bodies [10,11]. Several recent reports have shown that the knock-out or knock-down of RDR6 in plants did not only display more susceptibility to viruses, but also to bacterial and fungal pathogens, suggesting RDR6-mediated RNA silencing has a defense role against a wide range of pathogens including viral, bacterial and fungal pathogens [5,12,13,14,15,16]. Although SGS3 can also function in the pathway of trans-acting small-interfering RNA (ta-siRNA) [6], sense-RNA induced post-transcriptional gene silencing (S-PTGS) [5] or DNA virus-induced gene silencing (VIGS), SGS3 selectively defends against some RNA viruses [5,17,18], suggesting that the resistance of SGS3 to plant viruses is not a general phenomenon. For example, the deficiency of *SGS3* in *Arabidopsis* or oilseed rape (*Brassica napus*) conferred susceptibility to cucumber mosaic virus (CMV), but there was no obvious effect on turnip vein clearing virus (TVCV) in wild type *Arabidopsis* compared to *SGS3* mutant plants [5,18,19]. On the contrary, the accumulation of oilseed rape mosaic virus (ORMV) was positively correlated with expression levels of *SGS3* in oilseed rape [18].

Geminiviruses constitute a group of plant viruses with circular, single-stranded (ss) DNA genomes, which have a broad range of hosts and cause severe diseases in important crops worldwide [20]. *Begomovirus* is the largest genus of the Geminiviridae family, including 322 out of 369 species identified [21,22,23]. The genome of begomoviruses consists of one (monopartite) or two (bipartite) DNA molecules with the length of approximately 2.7 kb [21,22,23]. Monopartite begomoviruses are frequently found in associated with two classes of satellite DNA molecules (alphasatellite and betasatellite). These two satellites have no similarities to begomoviruses in genome organization except for the presence of a stem-loop structure that is required to initiate their replication [24]. Although geminiviral DNAs lack a dsRNA stage during their life cycle, they still could trigger RNA silencing and be targets of RNA silencing [17,25]. We have previously shown that *RDR6* in *N. benthamaina* (*NbRDR6*), an important component in the RNA silencing pathway, plays a pivotal role in defense against geminivirus infection [16]. However, it is still obscure whether *SGS3* in *N. benthamaina* (*NbSGS3*) also has a similar role to *NbRDR6* in RNA silencing pathway and anti-viral defense.

In this study, we show that NbSGS3 is required for induction and initiation of sense green fluorescent protein (GFP)-induced RNA silencing, and confirm the role of NbSGS3 in RNA silencing pathway. Furthermore, we show that NbSGS3 cooperates with NbRDR6 in triggering geminivirus-induced endogenous gene silencing (GIEGS) and in suppressing several geminivirus infections. These data offer insights into RNA silencing machinery–virus interaction in *N. benthamiana* plants, and provide valuable information for breeding resistant plants against viruses.

## 2. Materials and Methods

### 2.1. Plant Materials and Growth Conditions

*N. benthamiana* seedlings were placed in soil and incubated in an insect-free growth chamber at 25 °C and 60% relative humidity under a 16 h light/8 h dark photoperiod. Transgenic *Nicotiana benthamiana* GFP 16c and dsRDR6 lines were generous gifts of David C. Baulcombe (Cambridge University, UK).

### 2.2. Plasmid Construction

The construct pCHF3-35S-NbSGS3:Flag was described previously [11]. An RNAi construct containing an inverted repeat sequence of *NbSGS3* separated by an *Arabidopsis* intron was produced by overlapping PCR [26]. A fragment of the *NbSGS3* sense sequence was amplified using the primer pair A-SGS3-F/A-SGS3-intron-R, and overlapped with the intron sequence amplified using primer pair B-SGS3-intron-F/B-intron-R. The overlapping products were cloned into pCHF3 between the *Sac* I and *Bam*H I sites to produce pCHF3-35S:SGS3-intron. The corresponding antisense *NbSGS3* fragment was amplified using the primer pair C-SGS3-F/C-SGS3-R and subsequently cloned into pCHF3-35S:SGS3-intron between the *Bam*H I and *Sal* I sites to produce the RNAi construct pCHF3-35S:dsSGS3. An *NbRDR6* RNAi construct was generated in a similar manner. To construct a TRV-based recombinant VIGS vector containing *NbSGS3* or *NbRDR6*, a partial fragment of each gene was generated by PCR amplification using the respective primer pair and cloned into the pTRV2 vector (a kind gift of Yule Liu) [27] using the restriction enzyme sites listed in Table S1. The schematic diagrams of these constructs were shown in Figure S1. Binary vectors pCHF3-35S:GFP, pCHF3-35S:dsFP and pCHF3:p19 for PTGS suppression assays and pCHF3-35S:FP for S-PTGS have been described previously [16,28].

### 2.3. Plant Transformation

Transgenic lines over-expressing or down-regulating *NbSGS3* were obtained by transforming *N. benthamiana* with pCHF3-35S-NbSGS3:Flag or pCHF3-35S:dsSGS3, respectively. Transformation of *N. benthamiana* leaf discs, selection of transformants and transplantation of kanamycin-resistant shootlets have been described previously [16]. Transgenic plants were screened by PCR with specific primers targeted to promoter or intron sequences. Alterations in *NbSGS3* mRNA levels in transgenic plants were confirmed by RT-qPCR as described [16].

### 2.4. Viral Inoculation and Agroinfiltration

For PTGS experiments, transient silencing suppression assays were performed as described previously [29]. Classic two-component transient PTGS assays were performed by agroinfiltration of 35S:GFP with control or suppressor vectors into leaves of *N. benthamiana* 16c plants at the 5–6 leaf stage. For S-PTGS and IR-PTGS experiments, *Agrobacterium* cultures harboring pCHF3-35S:GFP, pCHF3-35S:FP and VSR-expressing vectors (for S-PTGS) or pCHF3-35S:GFP, pCHF3-35S:dsFP and VSR-expressing vectors (for IR-PTGS) were mixed in equal proportions and infiltrated into *N. benthamiana* leaves.

Geminivirus agroinoculation has been reported previously [11,30,31,32]. The tomato yellow leaf curl China virus (TYLCCNV)-derived VIGS vectors 2mDNA1 (pBinPLUS-2mDNA1) and 2mDNA1-NbSu (pBinPLUS-2mDNA1-NbSu) were constructed previously [33]. For the TRV-VIGS assay, *Agrobacterium* cultures harboring pTRV1 and pTRV2-VIGS (TRV2-GUS, TRV2-NbSGS3 or TRV2-NbRDR6) were mixed prior to inoculations, and then *Agrobacterium*-mediated inoculation onto *N. benthamiana* plants was performed as described previously [11].

### 2.5. DNA Isolation, DNA Blots Hybridization and DNA qPCR

DNA isolation, DNA blot hybridization and DNA qPCR were described previously [11,16,34,35].

### 2.6. RNA Extraction, Northern Blot, siRNA Blot and RT-qPCR Analysis

Total RNA was isolated from virus-infected plants and different plant organs using Trizol reagent (Invitrogen, Carlsbad, CA, USA). For Northern analyses of GFP mRNA, total RNA was separated by electrophoresis on a 1.5% formaldehyde gel, transferred to a Hybond-N^+^ membrane (GE Healthcare, Little Chalfont, UK), and hybridized with [α-^32^P-dCTP] labeled GFP probes using the Prime-a-Gene^®^ Labeling System (Promega, Madison, WI, USA). Hybridization signals were detected with a Typhoon 9200 imager (GE Healthcare, Little Chalfont, UK). For siRNA blotting and RT-qPCR analysis, the procedures have been described previously [16].

### 2.7. Protein Extraction and Western Blot Analysis

Total protein was extracted from infiltrated leaf patches (wild type or 16c *N. benthamiana* plants) or from the newly-emerged upper leaves of 16c *N. benthamiana* plants infected by TRV-VIGS as described previously [28]. Immunoblotting was performed with the GFP polyclonal antibody (ab6556), followed by goat anti-rabbit (ab6721) secondary antibody conjugated to horseradish peroxidase (Abcam, Cambridge, MA, US) as described [16].

## 3. Results

### 3.1. NbSGS3 Is Required for Induction and Initiation of Sense GFP-Induced RNA Silencing

**Figure 1:**
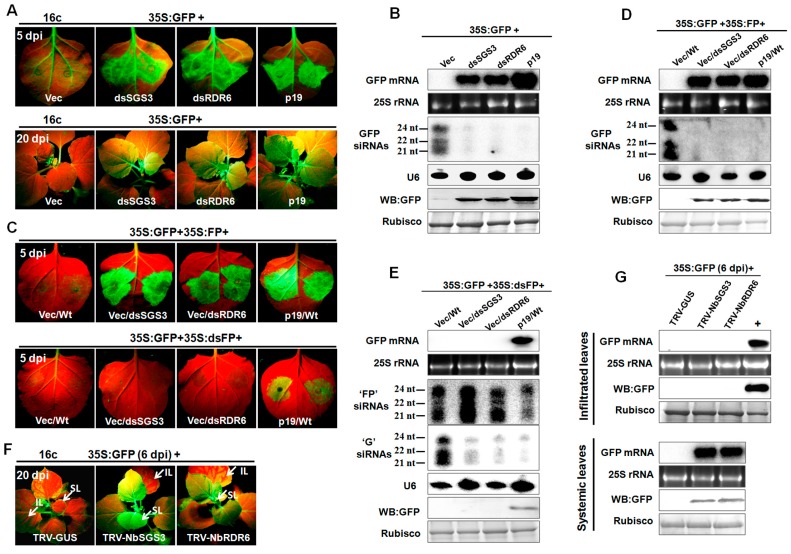
Reduced *Nicotiana benthamiana SGS3* (*NbSGS3*) expression suppresses sense-RNA induced post-transcriptional gene silencing (S-PTGS) and blocks the spread of systemic silencing. (**A**) Suppression of green fluorescent protein (GFP) silencing in *N. benthamiana* line 16c. Leaf patches were co-infiltrated with *Agrobacterium tumefaciens* cultures expressing GFP (35S:GFP) and either vector control (Vec), dsSGS3, double-stranded *Nicotiana benthamiana* RNA-dependent RNA polymerase (dsRDR6) or tomato bushy stunt virus (TBSV) p19. GFP fluorescence was photographed under UV light at 5 dpi (first row) and 20 dpi (second row); (**B**) accumulation of GFP mRNA, siRNAs and GFP protein in the infiltrated leaves shown in (**A**) at 5 dpi. For RNA blot analysis of mRNAs, [α-^32^P]-labeled DNA fragments of *GFP* were used as probes and ethidium bromide staining of 25S rRNA was used as a loading control. For small RNA blot analysis, [γ-^32^P]-labeled GFP or U6 oligonucleotides were used as probes. The size of the 21-, 22- and 24-nt RNAs are indicated. Protein levels were analyzed by immunoblot analysis using GFP monoclonal antibody. Coomassie blue staining of the large subunit of ribulose-1,5-bisphosphate carboxylase/oxygenase (Rubisco) served as a loading control; (**C**) GFP fluorescence in leaves of Wt, 35S:dsSGS3 or 35S:dsRDR6 transgenic *N. benthamiana* plants co-infiltrated with *Agrobacterium* cultures expressing 35S:GFP together with either a sense-PTGS trigger (35S:FP) (first row) or an inverted repeat of the GFP fragment (35S:dsFP) (second row) as indicated at the top of each panel. Plants were agroinfiltrated with 35S:GFP and the silencing triggers, together with vector (Vec) or p19 as indicated. Photographs were taken 5 dpi under UV light; (**D**,**E**) accumulation of GFP mRNA, siRNA and protein was detected in infiltrated leaves as described in (**C**). Labeled GFP oligonucleotides corresponding to the “FP”- and the “G”-regions of GFP were used to detect primary “FP”-siRNAs (1st siRNAs) and secondary “G”-siRNAs (2nd siRNAs), respectively; (**F**) function of NbSGS3 or NbRDR6 in initiation of systemic GFP silencing. 35S:GFP was infiltrated into 16c plants to induce local silencing, and after 6 days further infiltrated with TRV-GUS, TRV-NbSGS3 or TRV-NbRDR6. Photographs were taken 20 days post infiltration under UV light. Arrows indicate the infiltrated (IL) and systemic (SL) leaves; (**G**) accumulation of GFP mRNA and protein was detected in the infiltrated and systemic leaves as indicated in (**F**). “+” indicates RNA from a wild type 16c *N. benthamiana* leaf as a positive control. Coomassie blue staining of the large subunit of Rubisco served as a loading control.

To determine whether NbSGS3 plays a similar role to AtSGS3 in transgene-GFP-induced RNA silencing, a classic agroinfiltration assay using GFP-transgenic *N. benthamiana* 16c leaves was performed. *Agrobacterium tumefaciens* cultures harboring a binary vector capable of expressing a sense GFP mRNA (35S:GFP) and a hairpin RNAi construct containing *NbSGS3* (dsSGS3) or *NbRDR6* (dsRDR6) sequences under control of the 35S promoter of cauliflower mosaic virus (CaMV), were co-infiltrated into leaves of 16c plants. Agroinfiltration of 16c leaves with 35S:GFP and an empty vector (Vec) induced GFP RNA silencing, and led to reduced GFP fluorescence under UV light at 5 days post infiltration (dpi) (Figure 1A, top row). As expected, the intensity of green fluorescence increased substantially in leaf patches co-expressing GFP and tomato bushy stunt virus (TBSV) p19, which was used as a positive control for silencing suppression. Expression of dsSGS3 and dsRDR6 led to an increase in green fluorescence in co-infiltrated regions (Figure 1A, top row), indicating that silencing of *SGS3* and *RDR6* was able to suppress GFP-induced RNA silencing. Accordingly, RNA and protein gel blot analyses revealed that suppression of local silencing by dsSGS3 and dsRDR6 was accompanied by an accumulation of both GFP mRNA and protein, and loss of GFP-specific siRNAs in the infiltrated leaves (Figure 1B). The infiltrated plants were also monitored for the initiation of systemic silencing in upper young leaves under UV light. At 20 dpi, all plants infiltrated with empty vector showed the characteristic vein proximal GFP silencing in upper new leaves. In contrast, GFP fluorescence persisted in all leaves of 16c plants co-infiltrated with 35S:GFP plus dsSGS3, dsRDR6 or p19 (Figure 1A, bottom row), suggesting that a knock-down of either *NbSGS3* or *NbRDR6* expression compromises GFP-induced systemic silencing. These results demonstrated that NbSGS3 plays an important role in GFP-induced RNA silencing in 16c plants.

To determine whether NbSGS3 and NbRDR6 were required for S-PTGS and secondary siRNA amplification, their functions in suppression of S-PTGS and inverted repeat (IR)-PTGS were investigated. For S-PTGS, *Agrobacterium* cultures expressing 35S:GFP, 35S:FP (FP: a fragment of GFP) and empty vector were co-infiltrated into wild type (Wt), dsSGS3 and dsRDR6 transgenic *N. benthamiana* leaves. Leaves co-infiltrated with 35S:GFP, 35S:FP and p19 served as positive control. The IR-PTGS suppression assay was designed in a similar manner to that of S-PTGS, except that 35S:dsFP (dsRNA of FP) substituted for 35S:FP as the silencing trigger. As shown in Figure 1C, GFP expression in leaf patches co-infiltrated with vector in Wt *N. benthamiana* was silenced by expression of either the sense RNA trigger (35:FP) or the dsRNA inducer (35S:dsFP). Similar to the experiments shown in Figure 1A, silencing *NbSGS3* or *NbRDR6* (due to expression of dsSGS3 or dsRDR6) suppressed the GFP silencing triggered by 35S:FP, leading to bright green fluorescence in co-infiltrated areas of leaves (Figure 1C, top row). However, suppression was not observed when IR-PTGS was used as the silencing trigger, as the infiltrated areas showed no GFP signal in comparison to vector-infiltrated leaf patches (Figure 1C, bottom row). As a positive control, p19 suppressed both S-PTGS and IR-PTGS (Figure 1C). The presence of GFP fluorescence was further confirmed by immunoblot and RNA blot analyses (Figure 1D,E). GFP-specific siRNA blots showed that in comparison with Wt plants, *NbSGS3*- and *NbRDR6*-deficient plants produced drastically reduced, nearly undetectable levels of both GFP siRNAs (Figure 1D) and secondary siRNAs (“G” siRNAs) (Figure 1E) during S-PTGS, but showed no obvious changes in the accumulation of primary siRNAs (“FP” siRNAs) during IR-PTGS (Figure 1E). These data demonstrated that NbSGS3 and NbRDR6 are likely required for GFP-induced S-PTGS, but not for IR-PTGS.

We next examined whether NbSGS3 and NbRDR6 could also act in the initiation of systemic GFP silencing. *Agrobacterium* cultures harboring 35S:GFP were infiltrated into *N. benthamiana* line 16c and observed at 6 dpi for efficient induction of RNA silencing. Plants were then further agroinfiltrated with recombinant tobacco rattle virus (TRV) vectors carrying partial fragments of *NbSGS3* or *NbRDR6*. Knockdown of *NbSGS3* or *NbRDR6* was verified by RT-qPCR, which showed an approximate 80% reduction in the respective mRNA levels as compared to TRV-GUS-treated plants at 7 dpi (Figure S2). Twenty days after agroinfiltration with TRV recombinant constructs, systemic silencing of GFP was established in the TRV-GUS infiltrated plants, whereas intense GFP fluorescence was observed in plants infiltrated with TRV-NbSGS3 or TRV-NbRDR6. However, the infiltrated leaves still showed strong silencing when *NbSGS3* or *NbRDR6* expression was reduced (Figure 1F). Immunoblot and Northern blot analysis of GFP expression further supported this observation (Figure 1G). These data indicated that NbSGS3 and NbRDR6 were necessary for the initiation of systemic GFP silencing.

### 3.2. NbSGS3 Cooperates with NbRDR6 in Triggering Geminivirus-Induced Endogenous Gene Silencing (GIEGS) and in Suppressing Geminivirus Infection

**Figure 2:**
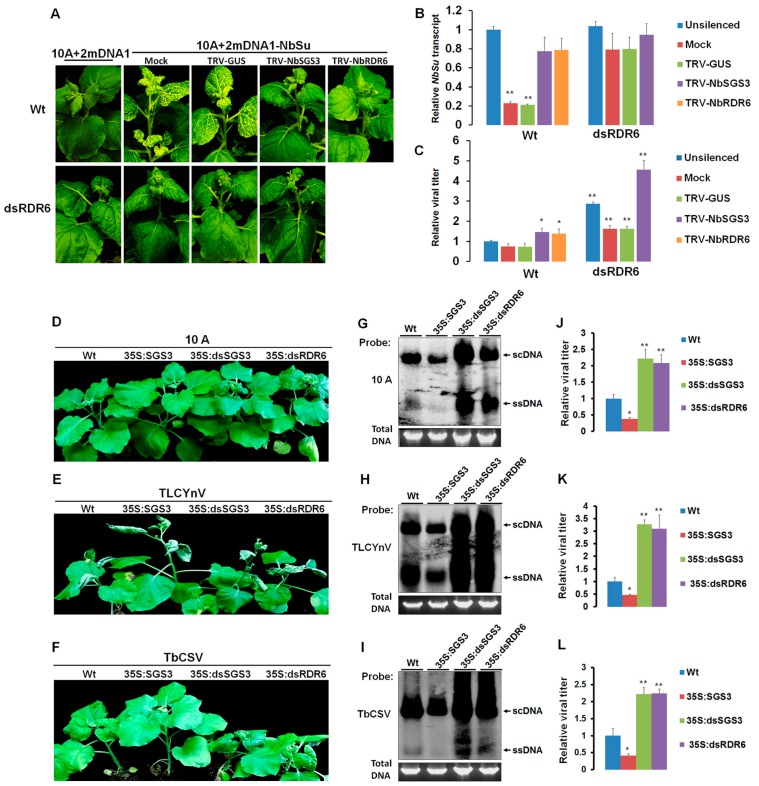
Compromised virus-induced gene silencing (VIGS) efficiency and enhanced susceptibility to geminivirus infection in *SGS3*-deficient *N. benthamiana*. (**A**) Wt or dsRDR6 transgenic *N. benthamiana* plants were mock inoculated or treated with TRV-GUS, TRV-NbSGS3 or TRV-NbRDR6, followed by infection with the 10A + 2mDNA1-NbSu VIGS vector. The phenotype exhibited by silencing of *NbSu* was observed at 30 dpi. Wt or dsRDR6 plants infected with an empty VIGS vector (10A + 2mDNA1) were used as non silenced controls; (**B**) RT-qPCR analysis of *NbSu* silenced plants. The relative levels of *NbSu* mRNA isolated from plants shown in (**A**) were normalized to mRNA of *NbGAPDH* that served as an internal standard. *NbSu* mRNA detected in Wt or dsRDR6 transgenic *N. benthamiana* plants infected by the empty VIGS vector (10A + 2mDNA1) represented levels present in non silenced plants. The relative level of *NbSu* mRNA in non silenced Wt plants is arbitrarily set as 1. Values represent the mean ± standard deviation (SD) from three independent experiments (*n* = 9). Student’s *t* test was performed to compare differences between unsilenced Wt and *NbSu* silencing plants and double asterisks indicate a highly significant difference (*p* < 0.01); (**C**) relative TYLCCNV (10A) DNA levels in plants shown in (**A**) which were normalized to 25S rRNA that served as an internal plant genomic DNA control. The relative level of 10A DNA in unsilenced Wt plants is arbitrarily set as 1. Values represent the mean ± standard deviation (SD) from three independent experiments (*n* = 9). Student’s *t* test was performed to compare differences between unsilenced Wt and *NbSu* silencing plants and double asterisks indicate a highly significant difference (*p* < 0.01); (**D**–**F**) symptoms induced by TYLCCNV(10A), TLCYnV or TbCSV when inoculated to Wt *N. benthamiana* plants or plants transgenic for 35S:SGS3, 35S:dsSGS3 or 35S:dsRDR6 at 20 dpi. Southern blot analyses (**G**–**I**) and qPCR (**J**–**L**) of geminivirus DNA accumulation in systemic leaves of the infected plants shown in (**D**–**F**). The relative level of viral DNA in infected Wt plants is arbitrarily set as 1. 25S rRNA levels served as an internal plant genomic DNA control. Values represent the mean ± standard deviation (SD) from three independent experiments (*n* = 9). Student’s *t* test was performed to compare differences between Wt and 35S:SGS3, 35S:dsSGS3 or 35S:dsRDR6 transgenic *N. benthamiana* plants, a single asterisk indicates a significant difference (*p* < 0.05) and double asterisks indicate a highly significant difference (*p* < 0.01).

The role of NbRDR6 in host antiviral defense against GIEGS and geminivirus infection has been reported [12,13,14,15,16]. To determine whether NbSGS3 has a similar role in defense against GIEGS or geminivirus infection, we first silenced *NbSGS3* or *NbRDR6* expression using TRV-induced gene silencing. The efficiency of *NbSGS3* or *NbRDR6* silencing was confirmed by RT-qPCR. Silenced plants were then inoculated with an established VIGS vector based on TYLCCNV (hereafter referred to as 10A) as the helper virus in association with a geminivirus alphasatellite derivative (2mDNA1) [33]. This leads to silencing of *NbSu* in plants inoculated with 10A + 2mDNA1-NbSu, displaying a yellow leaf color phenotype as a result of the inhibition of chlorophyll II biosynthesis. Typical phenotypes associated with Su-silencing developed in mock or TRV-GUS-treated plants, but not in *NbSGS3* or *NbRDR6*-silenced plants at 30 days post inoculation (dpi) (Figure 2A). To further investigate the efficiency of silencing, levels of *NbSu* mRNA in mock or TRV-GUS-treated plants silenced for *NbSGS3* or *NbRDR6* were measured using RT-qPCR. The data showed that expression of *NbSu* mRNA in mock or TRV-GUS-treated plants was less than 23% of levels detected in non-silenced control plants (infected by 10A + 2mDNA1) (Figure 2B). However, in *NbSGS3* or *NbRDR6*-silenced plants, expression of *NbSu* mRNA was about 80% of that in non-silenced control plants (Figure 2B). It is worth mentioning that helper viral DNA accumulated to higher levels in Wt plants silenced for *NbSGS3* than in mock or TRV-GUS-treated plants (Figure 2C). This is consistent with the plant being unable to mount an RNA silencing defense against TYLCCNV infection, rather than a failure to support virus infection.

To determine whether NbSGS3 has a general role in defense against geminivirus infection, transgenic *N. benthamiana* plants which either overexpressed (35S:SGS3) or silenced *NbSGS3* (35S:dsSGS3) were generated by *Agrobacterium*-mediated leaf disc transformation. Although transcription levels of *NbSGS3* varied in individual plants, the variation did not cause any detectable phenotypic changes (Figure S3). TYLCCNV (10A), tobacco curly shoot virus (TbCSV) and tomato leaf curl Yunnan virus (TLCYnV) were inoculated onto Wt, 35S:SGS3, 35S:dsSGS3 and 35S:dsRDR6 transgenic *N. benthamiana* at the 5–6 leaf stage. All three viruses caused more severe symptoms in transgenic plants expressing dsSGS3 and dsRDR6, but weaker symptoms in plants overexpressing *NbSGS3*, as compared to Wt plants (Figure 2D–F). Southern blot analyses (Figure 2G–I) and real-time quantitative PCR (qPCR) (Figure 2J–L) showed that a ~2-fold increase of 10A and TbCSV DNA levels and a ~3-fold increase of TLCYnV DNA accumulations in transgenic plants silenced for *NbSGS3* (dsSGS3) or *NbRDR6* (dsRDR6) as compared to Wt plants. In contrast, less than 50% viral DNA accumulations of Wt plants were detected in transgenic plants overexpressing *NbSGS3* (35S:NbSGS3). Based on the observation that overexpression of *NbSGS3* alleviated geminivirus symptoms and deficiency of *NbSGS3* promoted viral infection, we concluded that NbSGS3 plays an important role in host defense against geminivirus infection.

**Figure 3:**
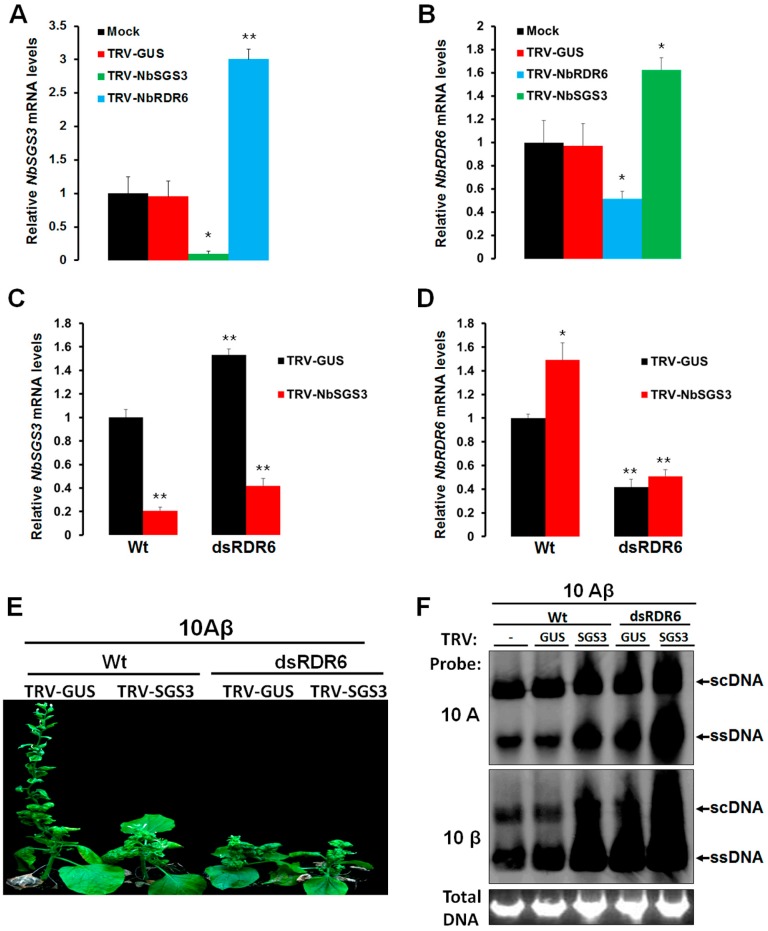
Confirmation of *NbSGS3* and *NbRDR6* silencing efficiency, and enhanced TYLCCNV/TYLCCNB infection. (**A,B**) mRNA levels in *N. benthamiana* leaves silenced for *NbSGS3* or *NbRDR6* after mock inoculation or TRV-GUS, TRV-NbSGS3 and TRV-NbRDR6 infection were analyzed by RT-qPCR using specific primers and normalized to *NbGAPDH* mRNA. Three independent experiments, each consisting of three mock or TRV-VIGS inoculated plants, were carried out for quantification analyses. The mRNA level of *NbSGS3 or NbRDR6* in mock plants was arbitrarily set to 1. Values represent the mean ± standard deviation (SD). Student’s *t* test was performed to compare differences between mock and TRV-VIGS inoculated plants, a single asterisk indicates a significant difference (*p* < 0.05) and double asterisks indicate a highly significant difference (*p* < 0.01); (**C,D**) *NbSGS3 or NbRDR6* mRNA levels in Wt or dsRDR6 transgenic *N. benthamiana* plants after infection with TRV-GUS or TRV-NbSGS3 were analyzed by RT-qPCR using specific primers and normalized to *NbGAPDH* mRNA. Three individual plants were used for each of the measurements. The relative mRNA level of *NbSGS3* or *NbRDR6* from TRV-GUS infected Wt plants was arbitrarily set to 1. Student’s *t* test was performed to compare differences between TRV-GUS infected and TRV-NbSGS3 infected Wt plants, or TRV-GUS infected and TRV-NbSGS3 infected dsRDR6 plants, a single asterisk indicates a significant difference (*p* < 0.05) and double asterisks indicate a highly significant difference (*p* < 0.01); (**E**) symptoms in Wt or dsRDR6 transgenic *N. benthamiana* plants treated with TRV-GUS, TRV-NbSGS3 (TRV-SGS3) followed by infection with 10Aβ at 40 dpi; (**F**) southern blot analyses of 10A and 10β accumulation in systemic leaves of the infected plants shown in (**E**) and mock plants (-) infected with 10Aβ at 40 dpi. Agarose gels were stained with ethidium bromide as a loading control. Viral single-stranded (ssDNA) and supercoiled (scDNA) DNA forms are indicated.

Interestingly, analysis of *NbSGS3* and *NbRDR6* mRNA levels in silenced plants showed that when *NbSGS3* expression was knocked down, expression of *NbRDR6* was unexpectedly enhanced. Similarly, expression of *NbSGS3* was higher when *NbRDR6* was down-regulated (Figure 3A,B). To determine whether NbSGS3 and NbRDR6 were able to cooperate in triggering GIEGS, TRV-GUS or TRV-NbSGS3 were inoculated onto transgenic plants silenced for *RDR6* (dsRDR6), and subsequently inoculated with 10A + 2mDNA1-NbSu. Relatively efficient silencing of *NbSGS3* and *NbRDR6* was observed (Figure 3C,D). As shown in Figure 2A,B, 10A-derived *NbSu* silencing was totally blocked in plants deficient for both *NbSGS3* and *NbRDR6*, and the helper viral DNA accumulated to higher levels than in plants deficient for *NbSGS3* or *NbRDR6* alone (Figure 2C). In addition, infection of both *NbSGS3* and *NbRDR6*-silenced plants by TYLCCNV and its associated betasatellite (hereafter referred to as 10Aβ) induced much more severe symptoms and accumulated higher levels of viral DNA than that in mock, *NbSGS3* or *NbRDR6*-silenced plants (Figure 3E,F). These results suggested that NbSGS3 and NbRDR6 have a synergistic relationship in triggering GIEGS and in suppressing geminivirus infection.

## 4. Discussion

RNA silencing is a major defense mechanism against foreign genes or viral invasion in plants [1,2,3]. However, plant viruses counteract this host defense mechanism by encoding viral suppressors of RNA silencing (VSRs) to promote their infection in plants. Almost all plant viruses encode their VSRs, and these VSRs act at different steps of RNA silencing pathway with various strategies to ensure their successful systemic invasion of specific hosts [36]. As the partner of RDR6, SGS3 can stabilize the 5’ overhang of dsRNAs, prevent their degradation, and recruit them for facilitating RDR6-mediated dsRNA synthesis [7,8,9,10]. Recent reports have shown that that SGS3 could be targeted by several VSRs or endogenous RNA silencing suppressor [11,37,38,39,40,41,42]. We showed NbSGS3 is an essential component of PTGS and is closely associated with geminivirus resistance in *N. benthamiana*. First, the knockdown of *NbSGS3* suppressed GFP-induced PTGS in 16c transgenic *N. benthamiana* plants (Figure 1A,B). Second, NbSGS3 was required for GFP-induced S-PTGS and secondary siRNA amplification, but not for IR-PTGS (Figure 1C–E). Third, NbSGS3 could also act in the initiation of systemic GFP silencing (Figure 1F–G). Forth, NbSGS3 was required for GIEGS and in suppressing geminivirus infection (Figure 2A,B). Fifth, NbSGS3 cooperates with NbRDR6 against GIEGS and geminivirus infection (Figure 2C–L). These data showed the role of NbSGS3 in RNA silencing and resistance to geminiviruses.

Plants with the knock-out of *AtSGS3* or the knock-down of *NbSGS3* (Figure 2) were more susceptible to several geminiviruses [17]. However, the resistance of SGS3 to RNA viruses is discrepant. In *Arabidopsis*, the sgs3 mutants exhibited enhanced susceptibility to CMV or CMV 2b deficient mutant but not to turnip mosaic virus (TuMV) or TVCV [5,18,19]. Because dsRNA mediated PTGS pathway, ssRNA-mediated PTGS pathway, miRNA-mediated RNA silencing and ta-siRNA mediated RNA silencing are closely linked in degrading exogenous RNA and regulating endogenous gene expression by sharing some common RNA silencing components. Therefore, it is possible that the specifically activated or repressed component by biotic and abiotic stress responses may breakdown the homeostasis of other pathways. For example, soybean mosaic virus (SMV) strain G7 infection induced the overexpressed AGO1 mRNA to trigger the AGO1 siRNA-mediated AGO1 mRNA degradation pathway, resulting in the reduced AGO1 level, which requires SGS3, RDR6 andSDE5 as well as DCL4/2 [43]. Therefore, there is a lower level of SMV strain G7 RNA in the *Glycine max SGS3* (*GmSGS3*)-silenced soybean than non-silenced plants [43], suggesting the complex relationship between RNA silencing components and hierarchical action of RNA silencing in defending viral infection. In addition, the involvement of VSRs would make these pathways be more complex and connected. For example, HC-Pro protein encoded by potyviruses, is a highly effective VSR that not only suppresses silencing but can also reverse the established RNA silencing [44]. The potyvirues, TuMV, potato virus A (PVA) and SMV, are not susceptible to the plants with the deficiency of *SGS3* [19,39,43], suggesting that HC-Pro or other viral proteins could inhibit SGS3 function and block SGS3/RDR6-dependent RNA silencing pathways. In contrast, the accumulation level of CMV 2b deficient mutant or wild type CMV in *Arabidopsis* or wild type CMV oilseed rape was negatively related to the expression of *SGS3* [5,18,19], suggesting SGS3-mediated resistance still works during CMV infection.

Taken together, the diversity of SGS3-mediated resistance reflects the complexity and diversity of RNA silencing pathways, and these pathways may be more complex and connected when dealing with viruses with VSRs. However, SGS3-mediated S-PTGS and resistance to geminiviruses are evidence-based, which will provide valuable strategy for breeding geminivirus-resistant plants.

## Supplementary Materials

Supplementary materials can be found at www.mdpi.com/1999-4915/9/9/247/s1.
