# Supplementary material for: SGS3 Cooperates with RDR6 in Triggering Geminivirus-Induced Gene Silencing and in Suppressing Geminivirus Infection in Nicotiana Benthamiana
Source: Viruses. 2017 Sep 4;9(9):247. doi: 10.3390/v9090247 (PMC5618013; doi:10.3390/v9090247)
Supplement: Supplementary file 1 [file viruses-09-00247-s001.pdf]

Supplementary Information

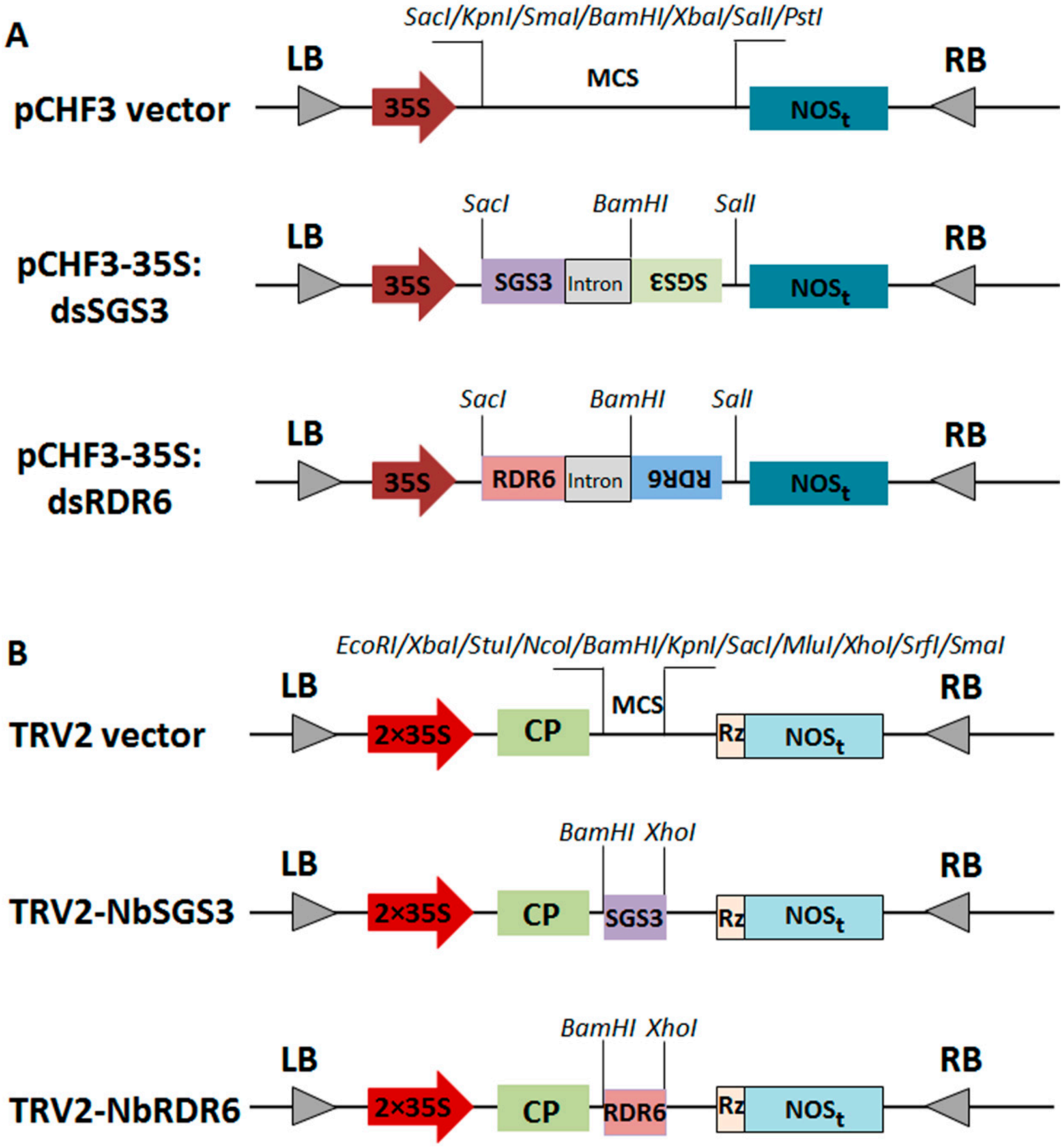

Figure S1. The schematic diagrams of constructs used in this study. (A) Schematic representation of RNAi constructs containing an inverted repeat sequence of *NbSGS3* or *NbRDR6* separated by an *Arabidopsis* intron. LB: left border, 35S: 35S promoter of cauliflower mosaic virus (CaMV), MCS: multiple cloning sites, NOS_t: termination sequence of the nopaline synthase gene; RB: right border. (B) Schematic representation of TRV-VIGS constructs carrying a partial fragment of *NbSGS3* or *NbRDR6* sequences. 2×35S: two tandem 35S promoters, CP: coat protein, Rz: ribozyme.

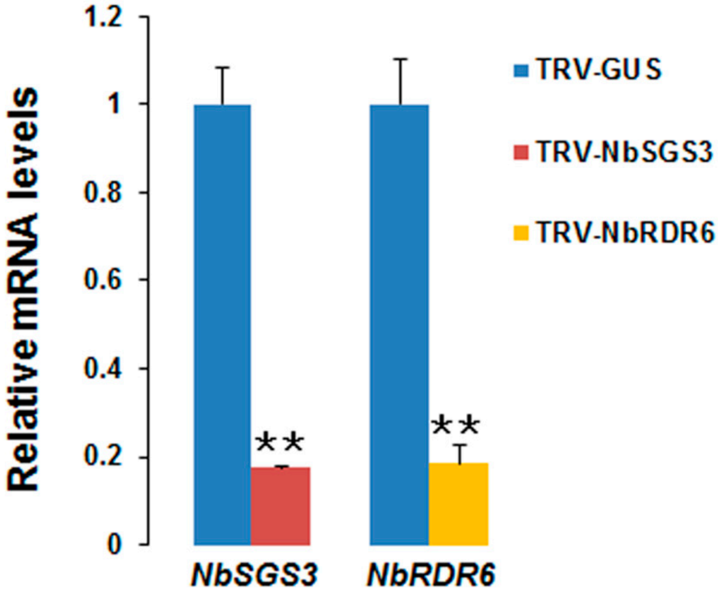

Figure S2. *The silencing efficiency of NbSGS3 or NbRDR6 in newly emerged leaves at 7 days post infiltration*. *NbSGS3* mRNA levels were reduced by approximate 83% in the plants infiltrated with *NbSGS3*-silencing vectors when compared to the plants infiltrated with TRV-GUS (control). *NbRDR6* mRNA levels were reduced by approximate 81% in the plants infiltrated with *NbRDR6*-silencing vectors when compared to the control. The mRNA level of *NbSGS3 or NbRDR6* in TRV-GUS infected plants was arbitrarily set to 1. Values represent means relative to the *NbSGS3* or *NbRDR6* levels in TRV-GUS infected plants. Double asterisks indicate $P < 0.01$ between the two treatments (Student's *t* test).

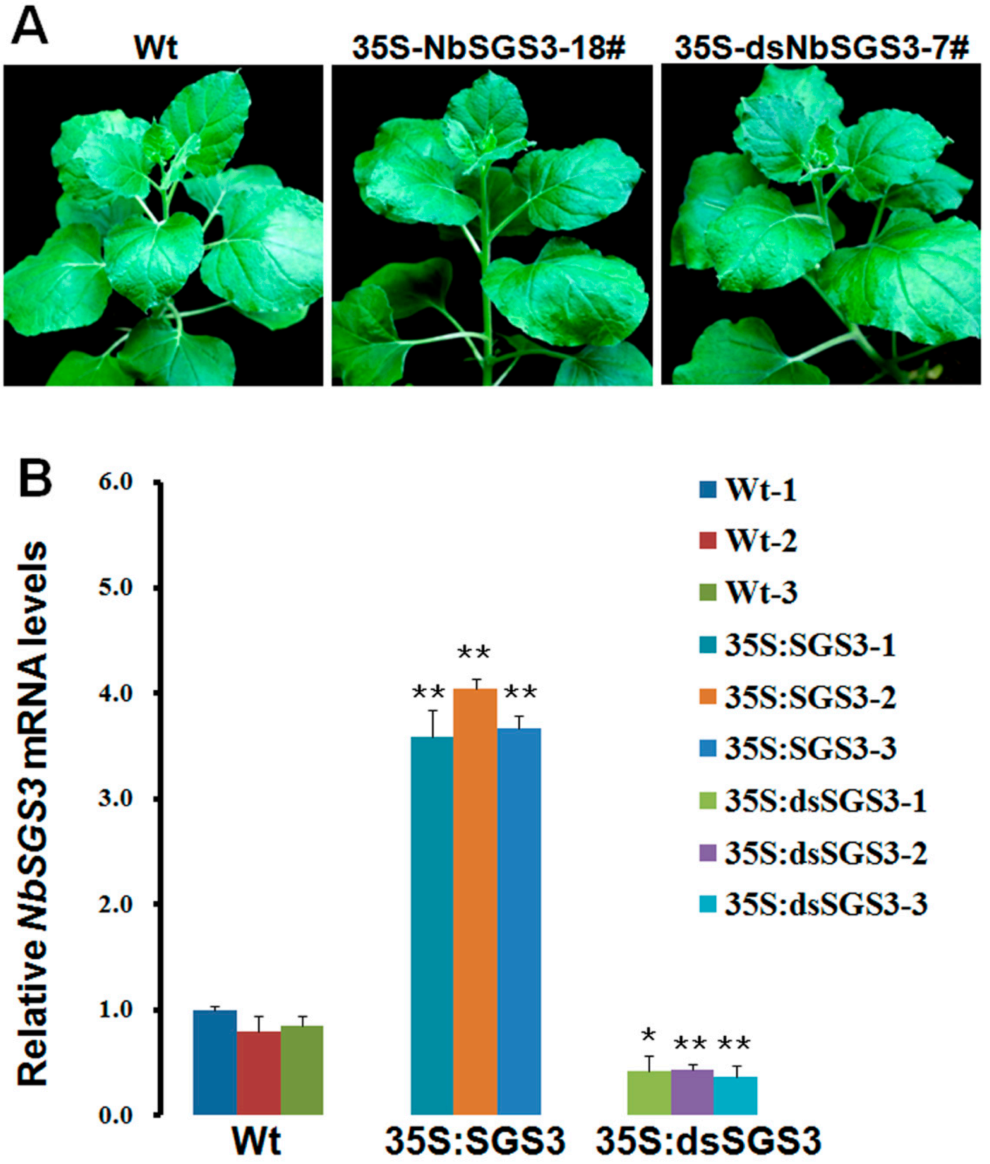

Figure S3. Ectopic expression or knockdown of *NbSGS3* in *N. benthamiana*. (**A**) Comparison of phenotypes in 40-day-old transgenic *N. benthamiana* plants overexpressing *NbSGS3* (35S-NbSGS3-18#) or silenced for *NbSGS3* (35S:dsNbSGS3-7#) and wild type (Wt) plants. (**B**) mRNA levels of *NbSGS3* were analyzed by RT-qPCR in 35S:SGS3 and 35S:dsSGS3 transgenic lines at the 6-7 leaf stage in the T1 generation. Three individual plants per phenotype (T1 generation) were used for each of the measurements. Values represent the mean ± standard deviation (SD). Student's *t* test was performed to compare differences between Wt plants and the transgenic lines, a single asterisk indicates a significant difference ($p<0.05$) and double asterisks indicate a highly significant difference ($p<0.01$).

Table1. Primers used in plasmid construction and other experiments in this study.

Primer Name	Primer Sequence (5'—3')	Purpose
NbSGS3:Flag-F	CGGGATCCATGAGTTCAAGCAAAGGGGTTG	pCHF3-NbSGS3: Flag, transient expression and transgene analysis
NbSGS3:Flag-R	GCGTCGACTTGAGATTGCTCTGGGGAG	
A-NbSGS3-F	TCCGAGCTCGAGATGAGGTCATACCAGGAAG	pCHF3-dsNbSGS3, transient expression, transgene
A-NbSGS3-Intro-R	ATCAGACTTACAACGTCCTCACACGATGATCC	
B-NbSGS3-Intro-F	GGATCATCGTGTGAGGACGTTGTAAGTCTGAT	pCHF3-dsNbSGS3, transient expression, transgene
B-Intron-R	CGGGATCCGCTCTATCTGCTGGGTCCAAATC	
C-NbSGS3-F	CGGGATCCCCTCACACGATGATCCTCAACC	pCHF3-dsNbSGS3, transient expression, transgene
C-NbSGS3-R	GCGTCGACGAGATGAGGTCATACCAGGAAG	
A-HpRDR6/Sac I/F	TCCGAGCTCGAAGAACAAGTTGGGCAAGTATGG	pCHF3-dsNbRDR6
A-HpRDR6+Intro/R	ATCAGACTTACAACGTCCAACCACAAAGTCATC	
B-HpRDR6+Intron/F	GATGACTTTGTGGTTGGACGTTGTAAGTCTGAT	pCHF3-dsNbRDR6
C-HpRDR6/BamH I/F	CGGGATCCCCAACCACAAAGTCATCTTTAC	
C-HpRDR6/Sal I/R	GCGTCGACGAAGAACAAGTTGGGCAAGTATGG	
Y10A-F	ATGTGGGATCCTCTGCTCAACGAGTTTC	To make probe for the detection of TYLCCNV in Southern blot
Y10A- R	CATCCTCAGACCTTGCGTTTCTTAAGAG'	
BETA01	GTAGGTACCACTACGCTACGCAGCAGCC	To make probe for the detection of TYLCCNB in Southern blot
BETA02	AGTGGTACCTACCCTCCCAGGGGTACAC	
TLCYnV-F	ATGCCTCGTCTTAATTCATTC	To make probe for the detection of TLCYnV in Southern blot
TLCYnV-R	TCAACTCTCCGTCGTCTGG	
TbCSV-F	ATCGATCTGGAAAATTCCATGATC	To make probe for the detection of TbCSV in Southern blot
TbCSV-R	GGATCCCACATAGTGCGGAG	
q-25S-rRNA-F	ATAACCGCATCAGGTCTCCA	As an internal control for relative quantitative genomic PCR analysis
q-25S-rRNA-R	CCGAAGTTACGGATCCATTT	
q-10A-F	TTAGAGATCGTCGTCCTAGTGG	Relative quantitative PCR analysis of TYLCCNV
q-10A-F	GCTCCTTACAAGCATATTGTCC	
q-10b-F	ATACATCATACTCATCCCCTACATCTA	Relative quantitative PCR analysis of TYLCCNB
q-10b-R	ATTATCCCACCATTCGACTTCAACATT	
q-TLCYnV-F	GGTGCGTCGCCGTCTGAACTTCG	Relative quantitative PCR analysis of TLCYnV
q-TLCYnV-R	CCAGTATGAGATACATCATGACGGGCC	
q-TbCSV-F	TACGCCGCCGTCTCAACTTCGAC	Relative quantitative PCR analysis of TbCSV
q-TbCSV-R	CTTTACCTATATGCTGAATGTCATGTCTGG	
35S-F	ACATGGTGGAGCACGACACG	PCR screening transgenic plants with CaMV 35S promoter
35S-R	GAGGAAGGGTCTTGCGAAGG	
TRV2-NbSGS3-F	CGGGATCCTCCAGCAAAGCAGATGATGG	TRV2-NbSGS3, the silencing of *NbSGS3*
TRV2-NbSGS3-R	CCGCTCGAGCATCTCGCGTTGCTCCTGC	
TRV2-NbRDR6-F	CGGGATCCGAAGAACAAGTTGGGCAAG	TRV2-NbRDR6, the silencing of *NbRDR6*
TRV2-NbRDR6-R	CCGCTCGAGCATCTGAAAATTTAAAGGGC	
NbGADPH-q-F	GCAGTGAACGACCCATTTATCTC	Relative qRT-PCR analysis of *NbGADPH*
NbGADPH -q-R	AACCTTCTTGGCACCACCCT	
NbSGS3-q-F	GGGCTCATATTAGGAAAGAAGCC	Relative qRT-PCR analysis of *NbSGS3*
NbSGS3-q-R	GTGAAAAGTATTTTGCATGCCCCG	
NbRDR6-q-F	CAGATTGAAAACAAAAATCCACCATG	Relative qRT-PCR analysis of *NbRDR6*
NbRDR6-q-R	ATTTTGCAACACGCTCTGCCC	
NbSu-q-F	5'-GCTTCTACACCCTTGTCTTCTCG-3'	Relative qRT-PCR analysis of *NbSu*
Nb-Su-q-R	5'-CCCCTATCACCCATTATCATCAC-3'	
